# Indigenous Ethnicity and Low Maternal Education Are Associated with Delayed Diagnosis and Mortality in Infants with Congenital Heart Defects in Panama

**DOI:** 10.1371/journal.pone.0163168

**Published:** 2016-09-20

**Authors:** Franz Castro, Julio Zúñiga, Gladys Higuera, María Carrión Donderis, Beatriz Gómez, Jorge Motta

**Affiliations:** Department of Research and Health Technology Assessment, Instituto Conmemorativo Gorgas de Estudios de la Salud, Panama City, Panama; TNO, NETHERLANDS

## Abstract

**Background:**

This is the first study in Panama and Central America that has included indigenous populations in an assessment of the association between socioeconomic variables with delayed diagnosis and mortality due to congenital heart defects (CHD).

**Methods:**

A retrospective observational study was conducted. A sample calculation was performed and 954 infants born from 2010 to 2014 were randomly selected from clinical records of all Panamanian public health institutions with paediatric cardiologists. Critical CHD was defined according to the defects listed as targets of newborn pulse oximetry screening. Diagnoses were considered delayed when made after the third day of life for the critical CHD and after the twentieth day of life for the non-critical. A logistic regression model was performed to examine the association between socioeconomic variables and delayed diagnosis. A Cox proportional hazards model was used to assess the relationship between socioeconomic features and mortality.

**Results:**

An increased risk of delayed diagnosis was observed in infants with indigenous ethnicity (AOR, 1.56; 95% CI, 1.03–2.37), low maternal education (AOR, 1.57; 95% CI, 1.09–2.25) and homebirth (AOR, 4.32; 95% CI, 1.63–11.48). Indigenous infants had a higher risk of dying due to CHD (HR, 1.43; 95% CI, 1.03–1.99), as did those with low maternal education (HR, 1.95; 95% CI, 1.45–2.62).

**Conclusion:**

Inequalities in access to health care, conditioned by unfavourable socioeconomic features, may play a key role in delayed diagnosis and mortality of CHD patients. Further studies are required to study the relationship between indigenous ethnicity and these adverse health outcomes.

## Introduction

Approximately, 10 per 1000 births are affected by congenital heart defects (CHD), of which a quarter is considered to be critical.[1,2] These defects constitute the leading cause of death among children with birth defects during the first year of life.[3] Despite this, around 13.8% to 29.5% of these infants receive a significantly delayed diagnosis.[4–6]

Previous studies have shown the association between socioeconomic factors with delayed diagnosis and mortality of CHD. Inequalities in access to health care seem to play a key role in these results.[7]

In Panama, Amerindian groups account for 11.6% of the population, and have been important contributors to the genetic diversity of the country.[8] These indigenous groups remain relatively isolated, in part due to geographical limits but mostly because of cultural barriers, and they are known to have disadvantaged socioeconomic conditions.[9] This is the first study done in Panama and Central America that has included indigenous populations in an analysis of the impact of socioeconomic factors on the disease outcomes of CHD. Very little is currently known about the influence of ethnicity on these outcomes.[10–12]

The objectives of this study are (1) to assess the association between socioeconomic variables and delayed diagnosis of CHD, and also (2) to explore the association between socioeconomic variables and mortality due to CHD.

## Methods

### Study Design, Inclusion and Exclusion Criteria

A retrospective observational study was conducted, including all public health care institutions with paediatric cardiologists in Panama. The cases included were born from January 1, 2010, to December 31, 2014, and had a diagnostic code from Q20.0 to Q26.9 according to the ICD-10 (International Classification of Diseases). All cases included were diagnosed by a paediatric cardiologist using echocardiography and Doppler studies with colour flow mapping. High resolution computed tomography or magnetic resonance imaging was used in some infants for a better characterization of the defects. Children with prenatal diagnosis were considered as diagnosed at birth for time to diagnosis analyses purposes. We excluded all cases of patent ductus arteriosus (PDA) born with less than 37 weeks, Marfan syndrome, congenital arrhythmias, cardiomyopathies, mitral valve prolapse and bicuspid aortic valve, based on reported definitions of CHD.[13,14]

### Definition of Critical Congenital Heart Defect

Infants were considered to have a critical congenital heart defect (CCHD) when diagnosed with at least one of the defects listed as primary or secondary targets of newborn pulse oximetry screening, such as dextro-transposition of the great arteries, truncus arteriosus, total anomalous pulmonary venous connection (TAPVC), tricuspid atresia, pulmonary atresia (with intact septum), hypoplastic left heart syndrome, tetralogy of Fallot, double-outlet right ventricle, Ebstein anomaly, coarctation/hypoplasia of aortic arch, aortic interruption/atresia/hypoplasia and single ventricle. Children with these twelve defects are likely to present with hypoxemia.[4,15]

### Definition of Outcome

Based on previous studies, the diagnosis of CCHD was considered delayed when made after the third day of life.[5,6] For the non critical congenital heart defect (NCCHD) group, the median of diagnostic times (20 days) was used as a cutoff value for defining delayed diagnosis. Sensitivity analyses were made using different values for the NCCHD group, observing similar results.

### Study Variables

Homebirth was defined as delivery outside a health facility. Maternal age was categorized in three groups according to legal adulthood age in Panama (18 years old) and the age for defining a high risk pregnancy (35 years old).[16] Low maternal education was defined as 6 years of education or less. Non-cardiac congenital anomalies were reported when major structural defects or syndromic chromosomal abnormalities were found.[4] Indigenous ethnicity was defined as meeting at least one of the following criteria: indigenous parental origin reported in the clinical record, place of residence or parental identity card number from an indigenous territory.

The Institutional Review Boards of the participating paediatric centers that authorized the analysis of clinical records approved the research protocol. We used identity card numbers instead of infants’ names to guarantee confidentiality of data.

### Data Sources

Institutional lists from the medical records departments were used to locate cases with CHD. Since those lists only contain infants’ identification and diagnosis, and Panama does not have electronic clinical files or national databases on CHD, all variables had to be obtained from paper based clinical records. Mortality from 2010 to 2014 was verified with the national database of mortality elaborated by the National Institute of Statistics and Census of Panama.

### Statistical Methods

An estimated 440 infants were diagnosed per year between 2010 and 2014, using information on live births from the National Institute of Statistic and Census and an estimated annual prevalence of 6.5 cases of CHD per 1000 live births for Latin America.[14] Sample calculation was performed using an expected frequency of delayed diagnosis of 29.5% and a confidence level of 95%.[6] Because of underdiagnosis due to a reduced amount of paediatric cardiologists and in order to guarantee a representative sample we preferred to use an estimate of diagnosed cases as the population size instead of institutional numbers of cases. We added an additional 25% to the sample, and a sample size of 1156 infants was obtained. After applying exclusion criteria (202 infants excluded), the final sample size was 954 infants. Using statistical software, we randomly selected infants from lists of health care institutions and then proceeded to locate their clinical files.

The frequency distributions of the most common defects and of socioeconomic variables were calculated and expressed as percentages. The presence of these variables was compared between indigenous and non-indigenous infants. These characteristics were also examined according to time of diagnosis (early or delayed).

### Logistic Regression Model

Univariate logistic regression models were used to estimate crude odds ratios (OR) for associations between each socioeconomic feature and delayed diagnosis. We also performed an adjusted logistic regression model, which included the following variables: maternal age, ethnicity, maternal education, delivery institution type, non-cardiac congenital anomalies, and severity of defects. Adjusted odds ratios (AOR) with 95% confidence intervals (CI) were calculated. The validity of the model was evaluated using an omnibus test (F test) with a significance level of 5%. The results are expressed as OR, AOR and 95% CI.

### Survival Analysis

Survival curves were made for the associations between each socioeconomic variable and mortality due to CHD and hazard ratios (HR) and their respective 95% CI were calculated using log-rank tests. An adjusted Cox proportional hazards model was performed to examine these associations, including maternal age, ethnicity, maternal education, delivery institution type, non-cardiac congenital anomalies, time to diagnosis (early or delayed) and severity of defects. Adjusted HR and 95% CI were also calculated. The model was evaluated using a likelihood ratio test.

All statistical analyses were assessed with IBM SPSS Statistics 19 and Graph Pad Prism 6 (La Jolla, CA).

## Results

From 2010 to 2014, we reviewed the clinical records of 954 infants with diagnosis of CHD. S1 Fig summarizes the most common defects found: VSD in 360 (37.7%), ASD in 184 (19.3%), PDA in 142 (14.8%) and TAPVC in 94 infants (9.9%).

### Socioeconomic Variables

Overall, low maternal education was documented in 258 (27%) infants and homebirth in 64 (6.7%) infants. A total of 217 (22.7%) infants belonged to an indigenous group. Among the indigenous infants, we observed that 126 (58.1%) had mothers with low education, 51 (23.5%) infants were born at home and 147 (67.7%) had a delayed diagnosis. Of the 737 non indigenous infants, 132 (17.9%) had mothers with low education, 13 (1.8%) infants were born at home and 356 (48.3%) had a delayed diagnosis (see Table 1).

**Table 1:** Socioeconomic features of infants with congenital heart defects in Panama according to ethnicity. 2010–2014.

Variables		General (n = 954)		Indigenous (n = 217)		Non-indigenous (n = 737)	
Maternal age (y)^a^		n	%	n	%	n	%
	< 18	73/954	7.7	18/217	8.3	55/737	7.5
	18–34	670/954	70.2	146/217	67.3	524/737	71.1
	35 or more	163/954	17.1	37/217	17.1	126/737	17.1
Ethnicity							
	Indigenous	217/954	22.7	-	-	-	-
	Non-indigenous	737/954	77.3	-	-	-	-
Maternal education^b^							
	Low	258/954	27	126/217	58.1	132/737	17.9
	High	541/954	56.7	51/217	23.5	490/737	66.5
Delivery institution type							
	Homebirth	64/954	6.7	51/217	23.5	13/737	1.8
	Health facility	890/954	93.3	166/217	76.5	724/737	98.2
Non-cardiac congenital anomalies							
	Yes	273/954	28.6	53/217	24.4	220/737	29.9
	No	681/954	71.4	164/217	75.6	517/737	70.1
Time to diagnosis							
	Early diagnosis	451/954	47.3	70/217	32.3	381/737	51.7
	Late diagnosis	503/954	52.7	147/217	67.7	356/737	48.3

Data are n (%), unless otherwise indicated; due to missing values percentages might not total 100. There were missing values for some variables:

^a^48,

^b^155

Table 2 shows the frequency of distribution of study variables by severity category and timing of diagnosis. CCHD was found in 345 (36.2%) cases and NCCHD in 609 (63.8%). Overall, 503 (52.7%) infants had a delayed diagnosis while 451 (47.3%) had an early diagnosis. For the CCHD group, 199 (57.7%) infants had a delayed diagnosis while for the NCCHD group, 304 (49.9%) infants had a delayed diagnosis.

**Table 2:** Socioeconomic features of infants with congenital heart defects in Panama according to congenital heart defect type (critical or non critical). 2010–2014.

Variables		CCHD (n = 345; 36.2%)			NCCHD (n = 609; 63.8%)			General (n = 954; 100%)		
		Total (n = 345; 100%)	Delayed diagnosis	Early diagnosis	Total (n = 609; 100%)	Delayed diagnosis	Early diagnosis	Total (n = 954; 100%)	Delayed diagnosis	Early diagnosis
			(n = 199; 57.7%)	(n = 146; 42.3%)		(n = 304; 49.9%)	(n = 305; 50.1%)		(n = 503; 52.7%)	(n = 451; 47.3%)
Maternal age (y)^a^		n	n (%)	n (%)	n	n (%)	n (%)	n	n (%)	n (%)
	< 18	27	15 (55.6)	12 (44.4)	46	25 (54.3)	21 (45.7)	73	40 (54.8)	33 (45.2)
	18–34	251	146 (58.2)	105 (41.8)	419	203 (48.4)	216 (51.6)	670	349 (52.1)	321 (47.9)
	35 or more	55	31 (56.4)	24 (43.6)	108	50 (46.3)	58 (53.7)	163	81 (49.7)	82 (50.3)
Ethnicity										
	Indigenous	88	70 (79.5)	18 (20.5)	129	77 (59.7)	52 (40.3)	217	147 (67.7)	70 (32.3)
	Non-indigenous	257	129 (50.2)	128 (49.8)	480	227 (47.3)	253 (52.7)	737	356 (48.3)	381 (51.7)
Maternal education^b^										
	Low	109	76 (69.7)	33 (30.3)	149	96 (64.4)	53 (35.6)	258	172 (66.7)	86 (33.3)
	High	190	97 (51.1)	93 (48.9)	351	164 (46.7)	187 (53.3)	541	261 (48.2)	280 (51.8)
Delivery institution type										
	Homebirth	31	30 (96.8)	1 (3.2)	33	26 (78.8)	7 (21.2)	64	56 (87.5)	8 (12.5)
	Health facility	314	169 (53.8)	145 (46.2)	576	278 (48.3)	298 (51.7)	890	447 (50.2)	443 (49.8)
Non-cardiac congenital anomalies										
	Yes	71	33 (46.5)	38 (53.5)	202	78 (38.6)	124 (61.4)	273	111 (40.7)	162 (59.3)
	No	274	166 (60.6)	108 (39.4)	407	226 (55.5)	181 (44.5)	681	392 (57.6)	289 (42.4)

CCHD = critical congenital heart defects; NCCHD = non critical congenital heart defects. Data are n (%), unless otherwise indicated; due to rounding, percentages might not total 100. There were missing values for some variables:

^a^48,

^b^155.

Of the infants with CCHD born at home, 30 (96.8%) had a delayed diagnosis, while for those born in a health facility, 169 (53.8%) had a delayed diagnosis. Among infants with CCHD from indigenous groups, 70 (79.5%) had a delayed diagnosis compared to 129 (50.2%) in the non-indigenous group. In infants with CCHD and low maternal education, a delayed diagnosis was observed in 76 (69.7%) infants in comparison to those with high maternal education, in which 97 (51.1%) infants had a delayed diagnosis.

Among infants with NCCHD and homebirth, 26 (78.8%) had a delayed diagnosis while 278 (48.3%) born in a health facility had a delayed diagnosis. In infants with NCCHD and indigenous ethnicity, delayed diagnosis was observed in 77 (59.7%), in comparison to the non indigenous group in which 227 (47.3%) infants had a delayed diagnosis. For those with NCCHD and low maternal education, 96 (64.4%) had delayed diagnosis, while for those with high maternal education, 164 (46.7%) had a delayed diagnosis.

### Delayed Diagnosis

Table 3 presents results of models for the associations between socioeconomic features and delayed diagnosis. After adjusting for all variables, an increased risk of delayed diagnosis was observed in infants with indigenous ethnicity (AOR, 1.56; 95% CI, 1.03–2.37), low maternal education (AOR, 1.57; 95% CI, 1.09–2.25) and those born at home (AOR, 4.32; 95% CI, 1.63–11.48). Infants with non-cardiac congenital anomalies were at a decreased risk of delayed diagnosis (AOR, 0.53; 95% CI, 0.38–0.73). These same associations persisted when we used different values for defining delayed diagnosis in infants with NCCHD.

**Table 3:** Logistic regression model for the association between socioeconomic variables and delayed diagnosis in infants with CHD in Panama. 2010–2014.

Variables		Crude OR		Adjusted OR	
Maternal age (y)^a^		OR	95% CI	OR	95% CI
	< 18	Reference		Reference	
	18–34	0.82	(0.47–1.42)	1.07	(0.72–2.00)
	35 or more	0.91	(0.65–1.28)	0.96	(0.65–1.42)
Ethnicity					
	Non-indigenous	Reference		Reference	
	Indigenous	2.25	(1.63–3.09)	1.56	(1.03–2.37)
Maternal education^b^					
	High	Reference		Reference	
	Low	2.15	(1.58–2.92)	1.57	(1.09–2.25)
Delivery institution type					
	Health facility	Reference		Reference	
	Homebirth	6.94	(3.27–14.72)	4.32	(1.63–11.48)
Non-cardiac congenital anomalies					
	No	Reference		Reference	
	Yes	0.51	(0.38–0.67)	0.53	(0.38–0.73)
Severity of defect					
	Non critical congenital heart defect	Reference		Reference	
	Critical congenital heart defect	1.37	(1.05–1.79)	1.11	(0.82–1.51)

There were missing values for some variables:

^a^48,

^b^155

### Survival Analysis

**Fig 1:**
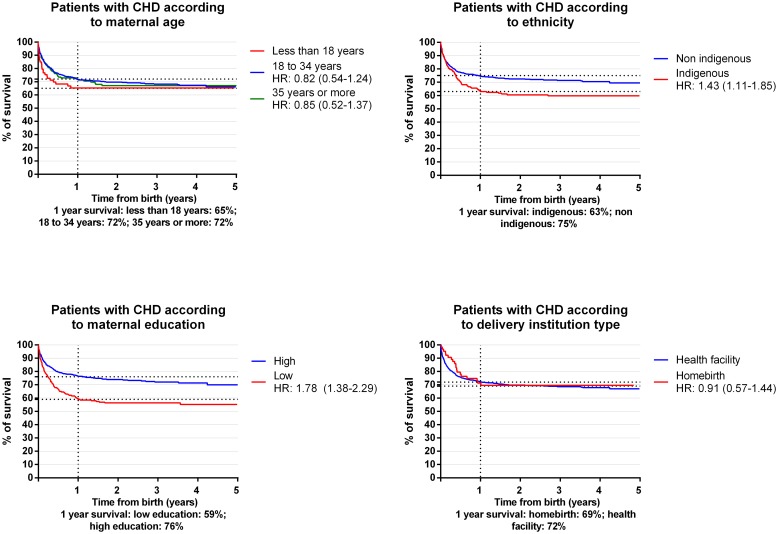
Survival curves for infants with congenital heart defects in Panama. 2010–2014.

From 2010 to 2014, 284 (30%) infants with CHD identified in our study died. Fig 1 shows survival curves and associations between socioeconomic variables and mortality due to CHD. Table 4 shows crude and adjusted HR for associations between socioeconomic variables and mortality due to CHD. For maternal age, a lower risk of dying was observed in infants born to mothers aged 18 to 34 years (HR, 0.82; 95% CI, 0.54–1.24) and in infants born to mothers aged 35 years or more (HR, 0.85; 95% CI, 0.52–1.37) in comparison to infants born to mothers less than 18 years old. The reported one year survival rate for infants born to mothers less than 18 years old was 65%, in comparison to 72% in infants born to mothers 18 to 34 years old and 72% in infants born to mothers aged 35 years or more. Regarding ethnicity, indigenous infants were more likely to die (HR, 1.43; 95% CI, 1.11–1.85) due to a CHD in comparison to non indigenous infants. We observed a one year survival rate of 63% for indigenous infants versus 75% for the non indigenous. When examining the association between maternal education and mortality, infants born to mothers with low education had a higher risk of dying (HR, 1.78; 95% CI, 1.38–2.29) in comparison to infants born to mothers with high education. The one year survival rate for the low education group was 59%, while it was 76% for the high education group. We did not observe any statistically significant associations between delivery institution type and mortality. Survival rates at one year were 69% for infants born at home and 72% for infants born in a health facility. When performing an adjusted model, infants at a higher risk of dying due to CHD were those that belonged to an indigenous group (HR, 1.43; 95% CI, 1.03–1.99) and those born to mothers with low education (HR, 1.95; 95% CI, 1.45–2.62).

**Table 4:** Cox proportional hazards model for the association between socioeconomic variables and mortality in infants with CHD in Panama. 2010–2014.

Variables		Crude HR		Adjusted HR	
Maternal age (y)^a^		HR	95% CI	HR	95% CI
	< 18	Reference		Reference	
	18–34	0.82	(0.54–1.24)	0.98	(0.62–1.54)
	35 or more	0.85	(0.52–1.37)	0.84	(0.50–1.40)
Ethnicity					
	Non-indigenous	Reference		Reference	
	Indigenous	1.43	(1.11–1.85)	1.43	(1.03–1.99)
Maternal education^b^					
	High	Reference		Reference	
	Low	1.78	(1.38–2.29)	1.95	(1.45–2.62)
Delivery institution type					
	Health facility	Reference		Reference	
	Homebirth	0.91	(0.57–1.44)	0.64	(0.38–1.10)
Non-cardiac congenital anomalies					
	No	Reference		Reference	
	Yes	1.74	(1.37–2.21)	2.16	(1.66–2.82)
Time to diagnosis					
	Early diagnosis	Reference		Reference	
	Late diagnosis	0.64	(0.51–0.81)	0.52	(0.39–0.68)
Severity of defect					
	Non critical congenital heart defect	Reference		Reference	
	Critical congenital heart defect	4.43	(3.46–5.67)	5.06	(3.86–6.64)

There were missing values for some variables:

^a^48,

^b^155

## Discussion

### Time to Diagnosis

Delayed diagnosis was found in 52.7% of the infants, a proportion that is approximately twice as high as the ones reported by previous studies.[4–6] A delayed diagnosis of CHD was associated with low maternal education, homebirth and belonging to an indigenous group, variables that are associated to poverty and reduced access to health care.

Previous studies have found an association between the levels of care received in hospitals by the newborn and delayed diagnosis in infants with CCHD. A retrospective study conducted in Massachusetts from 2004 to 2009, which evaluated 460 467 live births, found 916 cases of CCHD. In this study, a delayed diagnosis was found to be associated with delivery outside a tertiary care hospital (AOR, 3.6; 95% CI, 2.5–5.2).[5] Similar results were observed in another retrospective study done in Florida between 1998 and 2007, where 3603 cases with CCHD were analyzed. In this study there was a higher chance of having delayed diagnosis if the newborn received care at a level I nursery (AOR, 1.9; 95% CI, 1.6–2.2) or at a level II nursery (AOR, 1.5; 95% CI, 1.3–1.7) as compared to those that received care at a level III nursery.[4] We were not able to find studies that compared delays in diagnosis of CHD between infants born at home and infants born at hospitals, but we assume that there is a higher chance of delayed diagnosis in the former, especially in the setting of a low socioeconomic status.

The association between late detection of CHD with homebirth, indigenous ethnicity and low maternal education reflects the unfavourable socioeconomic status and multiple other disadvantages present in many indigenous communities in Panama and in many countries of the region. There are large disparities in distribution of health human resources (HHR) between indigenous and non-indigenous areas. A report by the Panamerican Health Organization recommends a minimum density of 25 health professionals per 10 000 population.[17] While in some regions of Panama this number is as high as 49.1 per 10 000 population, one of the most densely populated indigenous areas of Panama has the lowest HHR density in the country (2.2 per 10 000 population).[18]

Although we found studies reporting that some socioeconomic variables were factors associated with delayed diagnosis, [4,5] we were not able to find studies that showed a relationship between delayed diagnosis of CHD and indigenous ethnicity. We were only able to find a study conducted in Canada, which reported that there is a higher prevalence of CHD in indigenous populations.[19]

### Mortality

Infants that belonged to indigenous groups and were born to mothers with low education had significantly higher chances of dying due to a CHD, and also had lower one year survival rates. While an association between ethnicity and mortality has been found in studies done in the United States with African-American and white infants with CHD, [10–12] we were not able to find any studies describing an association between indigenous ethnicity and mortality from CHD.

There is a spectrum of determinants present in indigenous communities favoring poor health outcomes that goes beyond the prognostic and clinical factors associated with the severity of CHD. These factors encompass social, economic and cultural characteristics that condition the lower one-year survival rates found in the indigenous groups. Studies have shown the high costs and multidisciplinary approach required to provide optimal care for children with CHD.[20,21] Although we did not analyze the capacity of the Panamanian health system to deal with CHD, we believe that serious deficiencies in the public health care system and socioeconomic barriers are the main factors that keep many indigenous children with CHD from reaching adulthood.

Beyond this, we observed a high amount of indigenous infants with CHD, contributing to 22.7% of all the study cases while representing 11.6% of the country total population. Factors like exposure to teratogenic substances, consanguinity or genetic predisposition could be playing an important role on these findings.[22–26]

The main limitations of this study reside in the fact that we were unable to include all infants diagnosed with CHD in our analysis, due to a lack of national databases on congenital malformations. This study does not include data on missed diagnoses of CHD, and as a result we are lacking data on neonatal and infant mortality for those cases with missed diagnoses. Most of the infants were diagnosed at tertiary care facilities, which most likely led to a higher proportion of CCHD cases. We did not include infants treated at private hospitals because they give care to less than 15% of the population. Also, we did not include variables like income, maternal nutritional status and alcohol consumption because the data was unavailable. The main strengths of the study reside in the accuracy of the diagnoses, all made by paediatric cardiologists, and in the fact that all deaths were verified using a national mortality database.

Our results highlight the importance of social determinants in the management and outcomes of CHD. We believe that better access to quality health services should produce a positive impact on both diagnostic times and mortality.[7] The socioeconomic disparities that are revealed in this study are part of the contrasts seen in a nation with a GINI index of 51.7, [27] which has changed little in recent years despite a marked and sustained economic growth that has elevated Panama to the rank of an upper middle income country.[28]

To address these health deficits, Panama needs to strengthen the quality of cardiac care not only at regional levels but also at the level of local health care centers by increasing the quantity and quality of health care professionals capable of diagnosing and caring for infants with CHD.[29–31] Also, creating a surveillance system of birth defects would provide the epidemiological data needed to guide evidence-based national health policies regarding CHD.[32]

Our results suggest that inequalities in access to health care, conditioned by unfavourable socioeconomic features such as indigenous ethnicity, homebirth and low maternal education, may play a key role in the disease outcomes of CHD, specifically time to diagnosis and mortality. Further studies are required to study the relationship between indigenous ethnicity and these adverse health outcomes. We recommend that these indicators be strongly taken into consideration by health authorities when evaluating strategies for improving the quality of cardiac and paediatric health care, especially in countries with high levels of inequality and with an important quota of vulnerable groups such as indigenous populations.

## Supporting Information

S1 Fig Most common diagnoses found in infants with congenital heart defects in Panama. 2010–2014. VSD: ventricular septal defect; ASD: atrial septal defect; PDA: patent arterious ductus; TAPVC: total anomalous pulmonary venous connection; TGV: transposition of the great vessels; CAVC: complete atrioventricular canal defect. ^a^For PDA, only single defects were quantified.
(JPG)
