# Supplementary figures and images for: Indigenous Ethnicity and Low Maternal Education Are Associated with Delayed Diagnosis and Mortality in Infants with Congenital Heart Defects in Panama
Source: PLoS One. 2016 Sep 20;11(9):e0163168. doi: 10.1371/journal.pone.0163168 (PMC5029884; doi:10.1371/journal.pone.0163168)

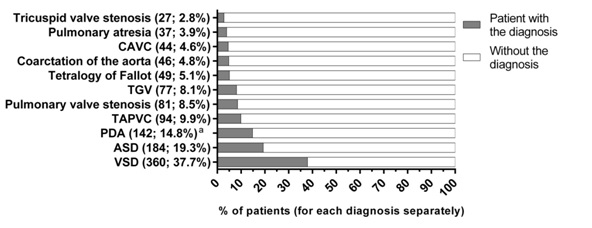

Supplement: S1 Fig — VSD: ventricular septal defect; ASD: atrial septal defect; PDA: patent arterious ductus; TAPVC: total anomalous pulmonary venous connection; TGV: transposition of the great vessels; CAVC: complete atrioventricular canal defect. aFor PDA, only single defects were quantified. (JPG) [file pone.0163168.s001.jpg]
